# What Method and Library Size Is Best for Fragment Screening?

**DOI:** 10.1002/cmdc.70503

**Published:** 2026-11-03

**Authors:** Stephen W. Fesik

**Affiliations:** ^1^ Departments of Biochemistry, Pharmacology and Chemistry Vanderbilt University Nashville Tennessee USA

**Keywords:** biology, chemistry, drug discovery

## Abstract

Fragment screening is widely used to provide useful starting points for drug discovery. There are many methods that are used today for conducting fragment‐based screens. and fragment libraries vary widely in size. In this perspective, I discuss the method that I use for fragment screening and the size of my fragment library, the reasons for these choices, and provide an example that illustrates the advantages.

## Graphical abstract

How to select the method for conducting a fragment screen and what size fragment library should you use?

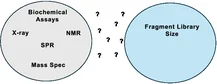

## 1 Introduction

Fragment screening has proven to be a useful tool in drug discovery. Since its inception 30 years ago [1], there have been many compounds discovered using fragment screening that bind to proteins, which have led to compounds that have entered clinic trials and are now approved drugs [2]. It is widely used in both industry and academia as a method for generating starting points in drug discovery programs. There are many advantages of the fragment‐based approach, including improved ligand efficiency, coverage of more chemical space, and by being smaller, fragments are easier to synthesize/purchase, less complex, and less likely to contain interfering moieties that block interactions with the protein [3].

Several methods have been used to identify fragment hits, such as NMR (both ligand‐observed and protein‐observed), X‐ray crystallography, surface plasmon resonance (SPR), differential scanning calorimetry, differential scanning fluorimetry (DSF), mass spectrometry, biochemical assays, and virtual screening. The choice of the method that is used to identify fragment hits varies widely, as evidenced from an analysis of the publications on fragment screening that have appeared in the last 10 years [2]. The most common approach was the use of biochemical assays followed by ligand‐observed NMR, SPR, and differential calorimetry. Compared to these methods, X‐ray crystallography, protein‐observed NMR, and virtual screening were less commonly used.

The size of the fragment libraries used to conduct fragment‐based screening also varies a lot, as shown in different studies. Fragment libraries can contain anywhere between 100 and 20,000 small molecules. There are many examples of the successful application of fragment screening using different screening methods and different‐size libraries. In this perspective, I discuss the method that I prefer to use for conducting fragment‐based screening, the size of the fragment library that I have chosen, and the reasons for these choices. I also present an example that illustrates the utility of this approach for finding fragment hits for Mcl‐1, a member of the Bcl‐2 family of proteins.

## 2 Methods for Conducting a Fragment Screen

The most common method used for identifying fragments that bind proteins is biochemical assays performed at high compound concentrations. This method has the advantage that the assays can be performed rapidly. However, no information is provided on how the compound binds to the target protein, and artifacts often arise in biochemical assays performed at high compound concentrations. There are also many biophysical methods that can be used for fragment screening, each with their own advantages and disadvantages. SPR is a popular method for fragment screening that requires immobilization of the target protein to a sensor chip. It doesn’t require a lot of protein, can quantitatively measure affinities, and can measure kinetics, but artifacts can be observed at high compound concentrations, and as such, SPR libraries have to be carefully constructed. No information is provided on where or how the ligand binds. Other methods for fragment screening, such as DSF and calorimetry, also have the limitation of not providing any structural information on ligand binding.

X‐ray crystallography is the method of choice for providing detailed structural information on how a ligand binds to a protein, and indeed, X‐ray crystallography has been used as a primary fragment screen. Although the throughput is slow, which has limited its widespread use, this has recently been dramatically improved and industrialized [4, 5]. In addition to the low throughput, however, there are other disadvantages of using X‐ray crystallography as a primary screen, such as fragment binding to many sites on the protein, binding affinity cannot be determined for each site, and false negatives arise from not being able to obtain the X‐ray structure of all protein/fragment complexes. Nevertheless, X‐ray crystallography has been used successfully to identify fragments that bind to proteins, and when combined with other techniques that can quantitatively measure binding affinities, it can be very effective [6].

NMR is used in fragment‐based screens in two ways. The most popular is ligand‐observed NMR in which ligand binding is detected by exploiting the difference in T2 relaxation/line broadening between the free and protein‐bound ligand. This method does not require the use of isotopically labeled protein and is not limited by the size of the protein. However, the false positives and false negatives obtained complicate the interpretation of the data, and no structural information is obtained on the protein binding sites.

The other NMR method for fragment screening is protein‐observed NMR in which ligand binding is detected by the chemical shift changes observed of the protein typically labeled with ^15^N or ^13^C. If the chemical shifts of the protein have been assigned, the general location of the binding site can be determined. The assignments are not necessary, however, and the chemical shifts can still be useful for distinguishing between different binding sites. In addition, by measuring the changes in the chemical shifts of the protein as a function of the added amount of the fragment, binding constants can be obtained. Artifacts caused by ligand‐induced protein aggregation can be readily detected in the NMR spectra, and there are no other components in the samples that can complicate the interpretation. This method for detecting and measuring ligand binding is robust and reliable even for ligands that bind weakly in the mM range and is applicable for proteins up to 100 kDa. For smaller proteins (<30 kDa), a simple two‐dimensional ^1^H/^15^N correlation experiment on uniformly ^15^N protein is sufficient (Figure 1a). To obtain narrower lines in medium‐size proteins with a large number of signals, a ^1^H/^15^N TROSY experiment [10] can be performed (Figure 1b), and for even larger proteins, a ^1^H/^13^C HMQC experiment is used with proteins containing ^13^C‐labeled methyl groups for methionine, leucine, isoleucine, and valine [7] (Figure 1c). For these reasons, I prefer protein‐observed NMR for conducting fragment‐based screens, especially for challenging proteins that are difficult to drug. Although the method requires large amounts of isotopically labeled protein and a significant amount of time to acquire the two‐dimensional NMR data on high‐field NMR spectrometers, the approach is worth the effort due to the ability to obtain structural information, quantitative binding affinities, and generates less artifacts.

**FIGURE 1:**
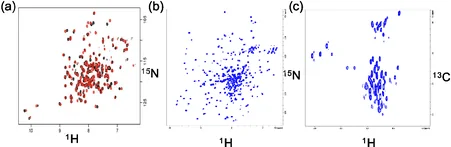
(a) ^1^H‐^15^N HSQC spectra of Mcl‐1 with (red) and without (black) a ligand [7], (b) ^1^H‐^15^N TROSY spectrum of the VCB complex [8], (c) ^1^H‐^13^C HMQC spectrum of ^13^C‐methyl labeled FEM1B [9]. (a) Reproduced with permission [11]. Copyright (2013), ACS.

## 3 Ideal Size of a Fragment Library

In my opinion, the size of the fragment library should be as large as possible and should be based on practical considerations. With a large library, many hits with similar structures are obtained, which can validate the hit and provide an initial assessment of the structure–activity relationships. Also, the chances of identifying molecules that bind to different sites and in different ways are increased. This can readily be obtained by the interpretation of the different chemical shits observed in the primary screen. This information is valuable for optimizing the compounds using a fragment merging or fragment‐linking approach. The use of larger libraries also lessen your chances of missing a key fragment hit that might not be present in a smaller library. By restricting the choice to only one example within a class of molecules due to size limitations, a compound that does not have a methyl group in the optimal position may be missed. Also, entire classes of molecules may not be present in a smaller library, such as an interesting heterocycle, which might have led to best fragment hit for follow‐up.

What are the practical considerations for choosing the size of a fragment library? The optimal library size should be the one that you can routinely screen in a reasonable amount of time for your needs. In my case, using protein‐observed NMR, I can screen a fragment mixture containing 12 compounds in 15 min (including changing the sample). Thus, I can test 96 fragment mixtures (1152 compounds, 1 × 96‐well plate) in a day and my entire library of 13,824 compounds (1152 fragment mixtures, 12 plates) in 12 days. Allowing for the preparation of the protein and obtaining the NMR time (cannot usually obtain continuous NMR time), the deconvolution of the fragment hits (assuming 30 hits), and the measurement of binding affinities, a fragment screen typically requires under 2 months to complete. For my needs, this is an optimal fragment library size based on the approach I have taken and the number of protein targets (6) that I screen in a year. Although doubling the size of the library to 27,648 compounds would theoretically be better, the time to acquire the NMR spectra would have to be reduced by half and the amount of isotopically labeled protein doubled (or even greater to obtain the signal‐to‐noise needed to acquire the NMR data in such short time). These considerations, along with the cost of the library, are important when deciding how big to make your fragment library.

## 4 Discovery of Mcl‐1 Inhibitors From Fragment Screening

Mcl‐1 is an anti‐apoptotic member of the Bcl‐2 family of proteins that is overexpressed and amplified in a variety of tumors and can prevent programmed cell death, keeping cancer cells aberrantly alive. Therefore, Mcl‐1 represents a promising therapeutic target for many cancers. In 2013, we reported [11] on an NMR‐based fragment screen using uniformly ^15^N‐labeled Mcl‐1, which illustrates the advantages of protein‐observed NMR and the use of a large fragment library containing 13,824 compounds. The screen was conducted using uniformly ^15^N‐labeled Mcl‐1 and yielded 132 hits of 11 distinct chemical classes. Based on the magnitude of the chemical shift changes, the most potent compounds were identified, which was confirmed in a fluorescence polarization assay by the *K* *_i_* values that were quantified. Two classes of fragment hits were followed up based on their affinities and the different but overlapping chemical shift changes caused by these two classes of fragments. These results suggested that we might employ a fragment merging approach for compound optimization. The class I fragment hits consisted of 6,5‐fused heterocyclic carboxylic acids. However, as shown in Table 1, the parent benzothiophenes, benzofurans, or indole‐2‐carboxylic acids were not identified in our initial fragment screen. It was only those compounds decorated with one or two Cl and/or Me substitutions could be detected in the fragment screen. Thus, the use of a smaller fragment library that only contained the parent molecules to represent this class of molecules would have missed these very important hits.

**TABLE 1:** SAR of fragment hits for binding to Bcl‐2 family proteins.

**S** **tructure**	Compd	Substitution		***K*** **_*i*_ , ** ‍**μM**		**LE** **(Mcl‐‍1)**
			Mcl‐1	Bcl‐‍xL	Bcl‐2	
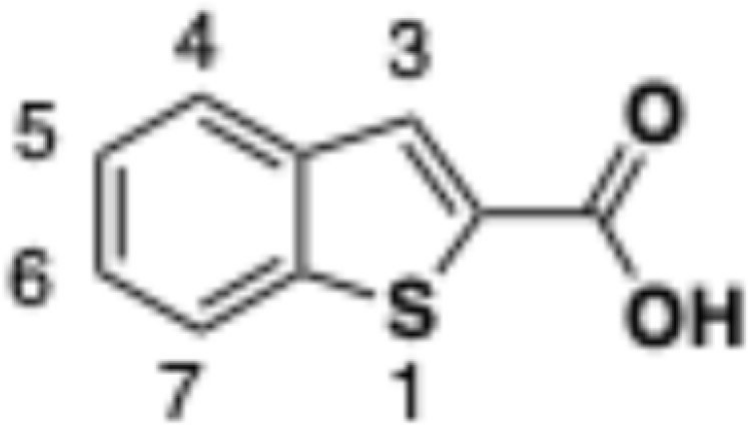	**1**	H	>1000			
	**2**	3‐Cl	131			**0.42**
	**3**	3,4‐di‐Cl	22	>1000	>1000	**0.46**
	**4**	3,6‐di‐Cl	59			**0.42**
	**5**	3‐Cl‐6‐Me	40	230	>1000	**0.43**
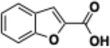	**6**	H	>1000			
	**7**	3‐Me‐5‐Cl	52			**0.46**
	**8**	3‐Me‐6‐Cl	23			**0.41**
	**9**	3,7‐di‐Me	102	>1000	>1000	**0.41**
	**10**	3,6,7‐tri‐Me	214	>1000	>1000	**0.34**
	**11**	4‐Br‐ 6‐Cl	90			**0.41**
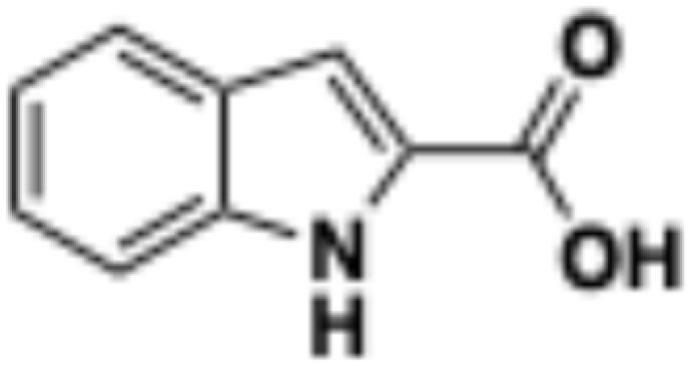	**12**	H	>1000			
	**13**	1‐Me	160			**0.41**
	**14**	1‐Me‐5‐Br	81			**0.41**
	**15**	3‐Ph‐7‐Me	136	>1000	>1000	**0.28**

*Source:* Reproduced with permission [11]. Copyright (2013), ACS.

Based on the chemical shifts alone that were obtained in the fragment screen, the class II hits appeared to bind to a slightly different site. We could not determine an X‐ray structure of either class I or II compounds when bound to Mcl‐1. However, we could quickly develop a model that was consistent with NOE data and fit with the NMR chemical shift changes that were observed. Based on the NMR‐derived model, we used a fragment merging approach in which a compound from class I with a *K* *_i_* = 131 μM was merged with a compound from class II with a *K* *_i_* = 60 μM to yield a compound with a *K* *_i_* = 0.32  μM (Figure 2) [11]. After the potency of the molecules was improved through fragment merging, we could obtain X‐ray structures of these compounds when bound to Mcl‐1. The X‐ray structures were very important to further improve the potency (single‐digit picomolar) and drug‐like properties of the Mcl‐1 inhibitors [12, 13, 14, 15].

**FIGURE 2:**
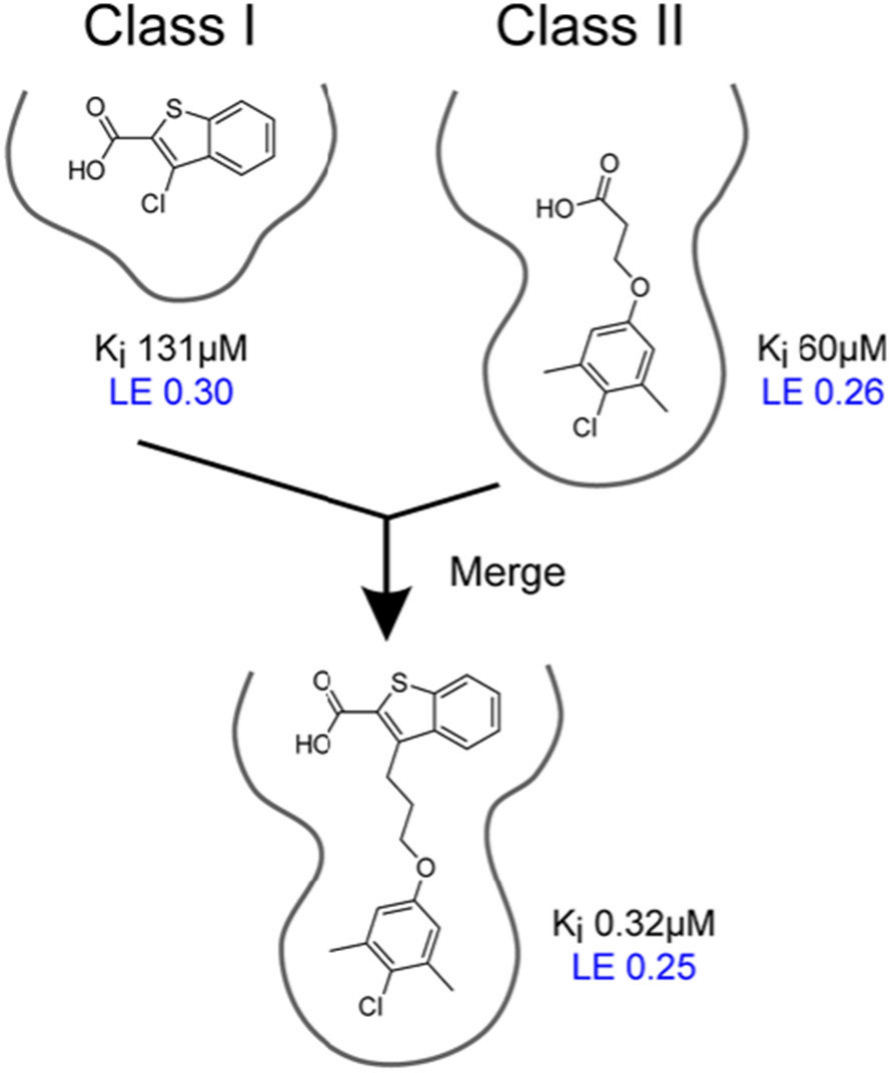
Schematic illustrating the merging of a class I and class II fragment to produce a potent Mcl‐1 inhibitor. Reproduced with permission [11]. Copyright (2013), ACS.

## 5 Conclusions

We have tried many methods for conducting fragment‐based screens over the past 30 years. In my experience, protein‐observed NMR is the method of choice for fragment screening and has had success in finding fragment hits even for proteins such as KRAS [16, 17] that were thought to be undruggable. In addition to the method that we used to conduct the screen, another key to our success is our relatively large fragment library.

In the future, it may be possible to conduct fragment‐based screens in less time to allow even larger fragment libraries to be screened. This may be accomplished by combining screening methods by first employing a faster method that does not give structural information, such as ligand‐observed NMR or a biochemical assay followed by one that does (e.g., protein‐observed NMR or X‐ray crystallography). Other advances in mass spectrometry [18], AI [19], cryo‐EM [20], photo‐hyperpolarized NMR [21], or ^19^F NMR [22] may also improve the way we conduct fragment screens.

## Disclaimer

The opinions expressed in this publication are the view of the author(s) and do not necessarily reflect the opinions or views of ChemMedChem, the Publisher, ChemistryEurope, or the affiliated editors.

## Conflicts of Interest

The author declares no conflicts of interest.

## Data Availability

The data that support the findings of this study are available on request from the corresponding author. The data are not publicly available due to privacy or ethical restrictions.
